# phippery: a software suite for PhIP-Seq data analysis

**DOI:** 10.1093/bioinformatics/btad583

**Published:** 2023-09-22

**Authors:** Jared G Galloway, Kevin Sung, Samuel S Minot, Meghan E Garrett, Caitlin I Stoddard, Alexandra C Willcox, Zak A Yaffe, Ryan Yucha, Julie Overbaugh, Frederick A Matsen

**Affiliations:** Computational Biology, Public Health Sciences Division, Fred Hutchinson Cancer Center, Seattle, WA 98109, USA; Computational Biology, Public Health Sciences Division, Fred Hutchinson Cancer Center, Seattle, WA 98109, USA; Data Core, Fred Hutchinson Cancer Center, Seattle, WA 98109, USA; Human Biology Division, Fred Hutchinson Cancer Center, Seattle, WA 98109, USA; Molecular and Cellular Biology Program, University of Washington, Seattle, WA 98195, USA; Human Biology Division, Fred Hutchinson Cancer Center, Seattle, WA 98109, USA; Human Biology Division, Fred Hutchinson Cancer Center, Seattle, WA 98109, USA; Molecular and Cellular Biology Program, University of Washington, Seattle, WA 98195, USA; Medical Scientist Training Program, University of Washington, Seattle, WA 98195, USA; Human Biology Division, Fred Hutchinson Cancer Center, Seattle, WA 98109, USA; Molecular and Cellular Biology Program, University of Washington, Seattle, WA 98195, USA; Medical Scientist Training Program, University of Washington, Seattle, WA 98195, USA; Human Biology Division, Fred Hutchinson Cancer Center, Seattle, WA 98109, USA; Department of Microbiology, University of Washington School of Medicine, Seattle, WA 98195, USA; Computational Biology, Public Health Sciences Division, Fred Hutchinson Cancer Center, Seattle, WA 98109, USA; Human Biology Division, Fred Hutchinson Cancer Center, Seattle, WA 98109, USA; Computational Biology, Public Health Sciences Division, Fred Hutchinson Cancer Center, Seattle, WA 98109, USA; Howard Hughes Medical Institute, Seattle, WA, 98109, USA

## Abstract

**Summary:**

We present the phippery software suite for analyzing data from phage display methods that use immunoprecipitation and deep sequencing to capture antibody binding to peptides, often referred to as PhIP-Seq. It has three main components that can be used separately or in conjunction: (i) a Nextflow pipeline, phip-flow, to process raw sequencing data into a compact, multidimensional dataset format and allows for end-to-end automation of reproducible workflows. (ii) a Python API, phippery, which provides interfaces for tasks such as count normalization, enrichment calculation, multidimensional scaling, and more, and (iii) a Streamlit application, phip-viz, as an interactive interface for visualizing the data as a heatmap in a flexible manner.

**Availability and implementation:**

All software packages are publicly available under the MIT License. The phip-flow pipeline: https://github.com/matsengrp/phip-flow. The phippery library: https://github.com/matsengrp/phippery. The phip-viz Streamlit application: https://github.com/matsengrp/phip-viz.

## 1 Introduction

Modern oligonucleotide synthesis allows researchers to generate highly multiplexed assays such as Phage Immunoprecipitation Sequencing (PhIP-Seq) (Mohan *et al.* 2018). This protocol is used to investigate linear epitopes by quantifying antibody-peptide interactions with phage-display libraries. These libraries may be general purpose, such as VirScan (Xu *et al.* 2015), or highly specialized as in Phage-DMS (Garrett *et al.* 2020). A typical PhIP-Seq dataset includes empirical serological or monoclonal antibody samples, negative controls by performing the protocol without antibodies present, and sequencing of the phage library to determine the input abundance of each peptide. The goal is to identify peptides in empirical samples that show robust enrichment due to antibody binding, relative to their expected abundances in the input phage library or no-antibody control.

Much of the published code for PhIP-Seq data analysis are specific to an experiment or task, forcing new researchers to piece together snippets from others or developing from scratch. Recently, Chen *et al.* (2022) published a R-based pipeline that includes enrichment quantification and plotting. The focus of our tool is to provide general infrastructure to standardize workflows and an API library applicable for any study design.

## 2 Design and usage

Here, we give brief descriptions of phip-flow, phippery, and phip-viz. The web documentation https://matsengrp.github.io/phippery/ provides more details, as well as instructions on how to contribute.

**Nextflow pipeline:** The phip-flow software is built using Nextflow (Di Tommaso *et al.* 2017) and presents end-to-end automation of reproducible workflows for PhIP-Seq data. The primary task of this workflow is quantification, normalization, and organized aggregation of results for any PhIP-Seq experiment starting from raw reads. Nextflow makes it easy to modify the pipeline, such as the use of alternate software for read alignment via editing template shell scripts with pre-defined inputs. Each step in the pipeline is run in a containerized environment, making it straightforward to add dependencies while maintaining workflow portability across computing platforms. By default normalization is done via edgeR (Robinson *et al.* 2010) although more sophisticated methods can be used (Chen *et al.* 2022). The inputs to the pipeline are comma-separated values (CSV) files specifying the sequencing data fastq files, the oligonucleotide sequences encoded in the phage library, and annotations for supplementary sample and peptide information. By default, alignment is performed with Bowtie2 (Langmead and Salzberg 2012), and the peptide counts are collated with SAMtools (Danecek *et al.* 2021). The workflow can easily scale to large sample sizes and handle any peptide library design.

**Figure 1.**
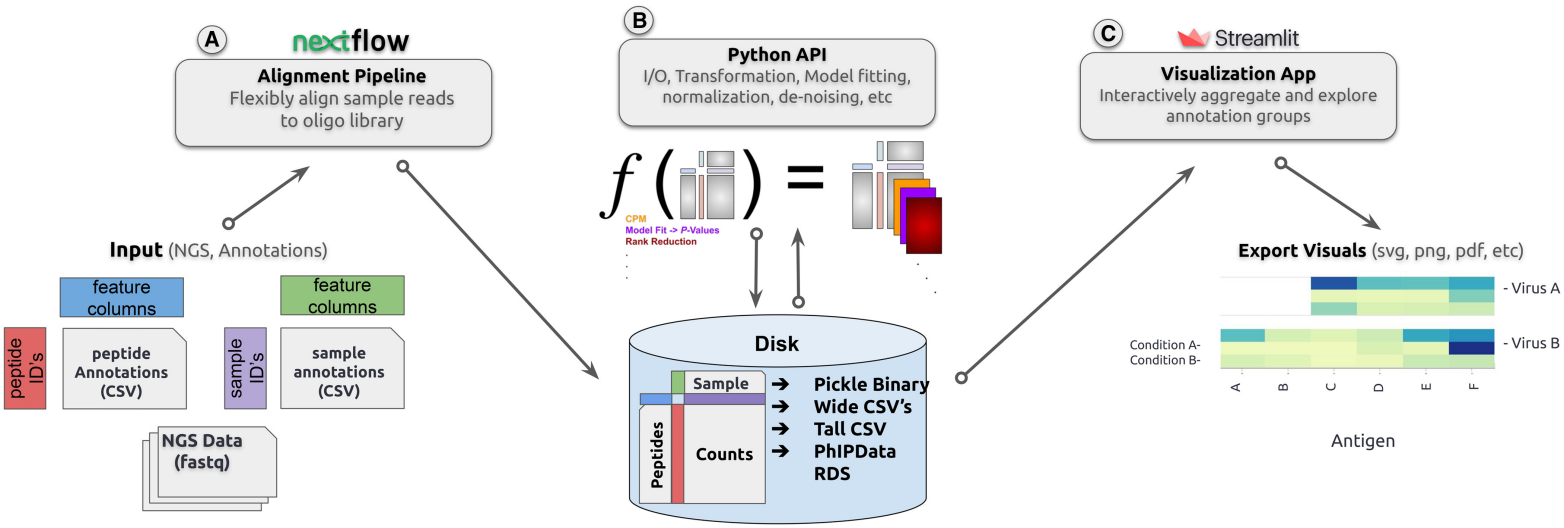
(A) The processing of sequencing data from a PhIP-Seq experiment begins with the alignment pipeline, which generates either a CSV file or xarray object containing the peptide counts across samples and associated annotations. (B) Custom analysis is performed with phippery library function calls, for example via the command line interface, and (C) visualization of results can be presented in an interactive manner using our Streamlit app.

**Python API:** The core data structure is a formatted xarray object (Hoyer and Hamman 2017), a multidimensional array well-suited for compiling the PhIP-Seq dataset into a single object to facilitate queries on the data. The phippery API imports commonly used data science dependencies and is designed to operate with a PhIP-Seq data xarray object. It is straightforward to build up an analysis with the phippery library of functions. Functions provided by the API include querying samples or peptides to a specific subset before exporting to CSV, essential tasks such as enrichment and Z-score calculations, as well as higher level analysis such as principal component analysis (Fig. 1). Several PhIP-Seq results have been published using phippery (Stoddard *et al.* 2021, Garrett *et al.* 2022, Willcox *et al.* 2022).

**Streamlit visualization:** A large majority of PhIP-Seq analysis tasks are simply aggregating samples from various treatment groups, and comparing their binding responses across peptide groups of interest in the phage library. For this, phip-viz may be used to explore subsets and group aggregations using widgets that provide insight into the dataset. This application takes as input only the binary formatted xarray dataset described above, and allows the user to subset data groups, aggregate, visualize, and export the resulting heatmaps.
